# The physical activity profile of active children in England

**DOI:** 10.1186/1479-5868-10-136

**Published:** 2013-12-16

**Authors:** Sarah Payne, Nick Townsend, Charlie Foster

**Affiliations:** 1 Department of Public Health, University of Oxford, Oxford, UK; 2 British Heart Foundation Health Promotion Research Group, Department of Public Health, University of Oxford, Old Road, Headington, Oxford OX3 7LF, UK

**Keywords:** ‘Physical activity’, England, Children ‘Active play’, Walking, Sport, Contributors, ‘Socioeconomic status’

## Abstract

**Background:**

In line with WHO guidelines, the UK government currently recommends that school-aged children participate in at least 60 minutes, and up to several hours, of at least moderate physical activity on a daily basis. A recent health survey indicates that the *amount* of reported physical activity varies by age, gender and socioeconomic status. The objective of this study is to identify what *types* of activity contribute most towards overall physical activity in children who achieve the UK physical activity recommendations; and how this varies according to age, gender and socioeconomic status.

**Methods:**

Self-reported physical activity was captured through the Health Survey for England 2008, a nationally representative, cross-sectional survey. We analysed data from 1,110 children aged 5–15 years who reported meeting the UK physical activity recommendations. The proportions of total physical activity achieved in various domains of activity were calculated and associations with age, gender and socioeconomic status were examined.

**Results:**

Active play was the largest contributor to overall physical activity (boys = 48%, girls = 53%), followed by walking (boys = 17%, girls = 23%). Active school travel contributed only a small proportion (6% for boys and girls). With increasing age, the contribution from active play decreased (rho = -0.417; p < 0.001) and the contribution of walking (rho = 0.257; p < 0.001) and formal sport (rho = 0.219; p < 0.001) increased. At all ages, sport contributed more among boys than girls. Sport contributed proportionately less with increasing deprivation (rho = -0.191; p < 0.001).

**Conclusions:**

The contributors to overall physical activity among *active* children varies with age, socioeconomic status and gender. This knowledge can be used to target interventions appropriately to increase physical activity in children at a population level.

## Background

Regular physical activity is associated with a range of health benefits for children including beneficial effects on musculoskeletal health and adiposity [1,2]; mental health and cardiovascular disease risk factors such as hypertension and high levels of high-density lipoprotein cholesterol (HDL-C) [2]. Participation in regular physical activity in childhood and adolescence has also been reported to positively influence physical activity levels in adulthood [3,4].

The UK government currently recommends that school-aged children participate in at least 60 minutes, and up to several hours, of at least moderate physical activity on a daily basis [5]. This recommendation is based on the current evidence and is similar to other national physical activity recommendations [2,6,7]. The 2008 Health Survey for England (HSE) reported that only 32% of all boys and 24% of all girls aged 2–15 years in England meet this recommendation [8]. This survey also identified differences in the amount of reported physical activity by age, particularly in girls, and by socioeconomic status. Differences in physical activity in children by age and gender have also been reported elsewhere in the literature [9-11], however the evidence of association with socioeconomic status is not clear [12,13].

There are various domains of activity that contribute to children’s overall physical activity levels including: active travel; organised or structured sport; informal play; domestic and leisure activities. The promotion of physical activity in both adults and children has been an important policy agenda in recent years. Current policies and strategies to increase physical activity in children promote a multi-component approach, across a variety of interventions in different settings [14-17]. A better understanding of the types of activity most commonly undertaken by *active* children could help target interventions toward specific groups of children, to have the most impact on increasing physical activity at a population level.

This study aims to identify what types of activity contribute most to the overall physical activity levels in children who reported achieving the UK recommendations; and how this varies according to age, gender and socioeconomic status.

## Methods

This study uses data from the 2008 Health Survey for England, a nationally representative, private-household based, cross-sectional survey of children and adults in England. The 2008 HSE included specific questions on physical activity in addition to the general health questions included in other years. The children’s physical activity questionnaire was validated in 2007 and found to have “moderate external validity for measuring the time spent in moderate or vigorous physical activity” [18].

A multi-stage, stratified, random, core sample of 16,056 addresses and a boost sample of 19,404 addresses were selected. Each individual within a selected household was eligible for inclusion. Where there were more than two children in a household, two were randomly selected for inclusion. Interviews were carried out in 64% of households in the general population sample and 73% of households in the boost sample yielding 3,473 children aged 0–15 years and 4,048 children aged 2–15 years respectively giving a total sample of 7,521 children.

Children aged 13–15 years were interviewed directly, whereas the parents of children aged 0–12 years were asked about their children, with the children present wherever possible. Children were asked to recall their physical activity for each day over the seven days prior to the interview day; excluding physical activity during school curriculum time. Intensity of physical activity was not measured and has not been used in this analysis.

Data on demographic and social factors were also collected including age, sex and socioeconomic status (measured by Index of Multiple Deprivation 2007 (IMD) quintile). The IMD is a composite score of seven domains: Income deprivation; Employment deprivation; Health deprivation and disability; Education, skills and training; Barriers to housing and services; Crime; The living environment, which are weighted and combined together to create the overall IMD. More detail on the HSE survey methodology can be found elsewhere [19].

In our analysis we included children aged 5–15 years (1,934 children aged <5 years were excluded). We excluded children who had not attended school on at least one day in the past seven days (n = 952) and children whose self-reported activity per day was less than zero minutes in any category of physical activity (n = 21).

Summary physical activity variables were derived from the data and activities were grouped into six categories based on those used in the HSE questionnaire: domestic activity (e.g. gardening & housework); active travel (walking or cycling to/from school only); walking (excluding to/from school); cycling (excluding to/from school); active play (e.g. skipping, general play, hopscotch, dancing); sport (including team sports such as rugby or hockey; individual sports; or structured activities such as swimming, gymnastics, yoga or horse riding).

### Statistical analyses

We calculated the mean minutes per week, across all children, for each category of physical activity and the percentage of mean total minutes of physical activity accounted for by each category. Data were weighted for child selection within each household using HSE weights [19] and analysed using STATA 11 [20]. Descriptive analysis investigated the contribution of each category of physical activity to total physical activity, stratified by demographic and socioeconomic factors. Associations between these factors and reported physical activity were tested using one-way ANOVA and trends were tested using Spearman’s Rank test.

Children were considered to have achieved the recommended level of physical activity if they reported at least 60 minutes of physical activity a day on all seven days of the week; in line with the 2011 UK Government recommendations.

## Results

After exclusions, 4,614 children remained in our sample for analysis, of which 24% (1,110) met the recommendation of 60 minutes or more of physical activity per day (19% of girls and 29% of boys). Active play contributed the most to total physical activity (50%), followed by walking (19%). A large proportion of children (58%) did sufficient active play to achieve their total weekly recommendation of physical activity through this activity alone; 18% achieved it through walking alone, whereas only 11% achieved it through sport alone.

### Gender

Stratified by gender, active play contributed 48% among boys; the next largest contributor was walking (17%) followed closely by sport and cycling (16% and 12% respectively). Active travel contributed only 6% and domestic activities such as housework and gardening contributed minimally (Table 1).

**Table 1:** Contribution of categories of physical activity among girls and boys by age and socioeconomic status (mean minutes per week; and mean percentage contribution to total weekly physical activity)

		**All physical activity**	**Sport**		**Active travel**		**Active play**		**Walking**		**Cycling**		**Domestic activity**	
		**Mins**	**Mins**	**%**	**Mins**	**%**	**Mins**	**%**	**Mins**	**%**	**Mins**	**%**	**Mins**	**%**
**Boys (n)**	All (668)	1,229	201	16	71	6	574	48	225	17	144	12	13	1
**Age group (n)**	5-6 yrs (123)	1,035	85	8	54	6	665	63	141	13	85	9	6	1
	7-8 yrs (118)	1,083	147	15	66	6	597	55	134	12	131	12	9	1
	9-10 yrs (131)	1,201	204	15	52	5	605	52	155	12	172	15	13	1
	11-12 yrs (118)	1,204	204	19	91	7	543	44	203	18	147	12	16	1
	13-15 yrs (178)	1,498	313	20	88	6	495	34	408	27	173	11	20	1
**Girls (n)**	All (442)	1,121	118	10	66	6	599	53	249	23	68	5	21	2
**Age group (n)**	5-6 yrs (97)	1,079	40	4	70	7	732	67	149	15	80	7	8	1
	7-8 yrs (93)	1,083	86	9	62	6	745	67	125	13	46	4	18	2
	9-10 yrs (93)	1,086	118	10	43	5	594	54	226	23	93	7	13	1
	11-12 yrs (74)	1,117	129	12	81	8	474	43	314	28	88	7	31	2
	13-15 yrs (85)	1,255	231	16	79	7	403	32	468	38	35	3	39	4
**Boys (n)**	All (668)	1,229	201	16	71	6	574	48	225	17	144	12	13	1
**IMD quintile (n)**	Least deprived (118)	1,059	212	20	59	6	457	44	217	20	103	9	11	1
	Q2 (109)	1,269	314	22	67	6	524	44	210	16	139	11	15	1
	Q3 (136)	1,211	153	13	87	7	589	50	225	18	148	12	9	1
	Q4 (139)	1,222	178	14	68	5	601	52	187	14	170	13	18	1
	Most deprived (166)	1,345	178	12	73	5	656	51	271	18	152	13	14	1
**Girls (n)**	All (442)	1,121	118	10	66	6	599	53	249	23	68	5	21	2
**IMD quintile (n)**	Least deprived (82)	991	141	14	56	6	496	50	207	22	72	6	18	2
	Q2 (75)	968	123	13	60	6	552	55	159	18	57	6	19	2
	Q3 (82)	1,064	123	9	58	6	543	53	248	23	80	7	14	2
	Q4 (86)	1,150	90	8	65	6	620	56	269	24	90	6	15	1
	Most deprived (117)	1,331	116	8	84	7	727	54	323	26	49	3	33	3

Among girls there was a slightly different pattern. Active play contributed slightly more than in boys (53%); as did walking (23%), with smaller contributions coming from sport (10%) and cycling (5%). Similarly with boys, active travel contributed 6% and domestic activities contributed a minimal amount.

### Age

**Figure 1:**
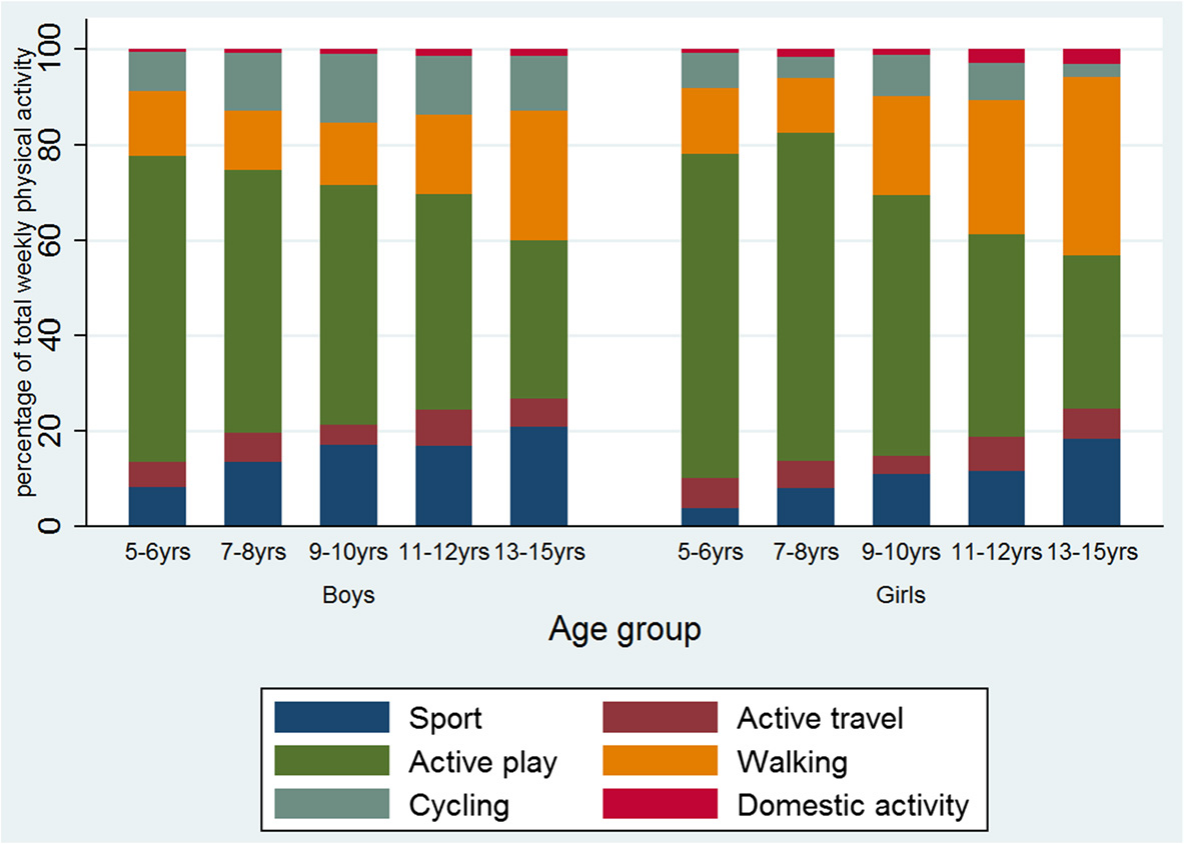
Mean percentage of all physical activity contributed by each category of activity, by age group and gender in England in 2008.

**Figure 2:**
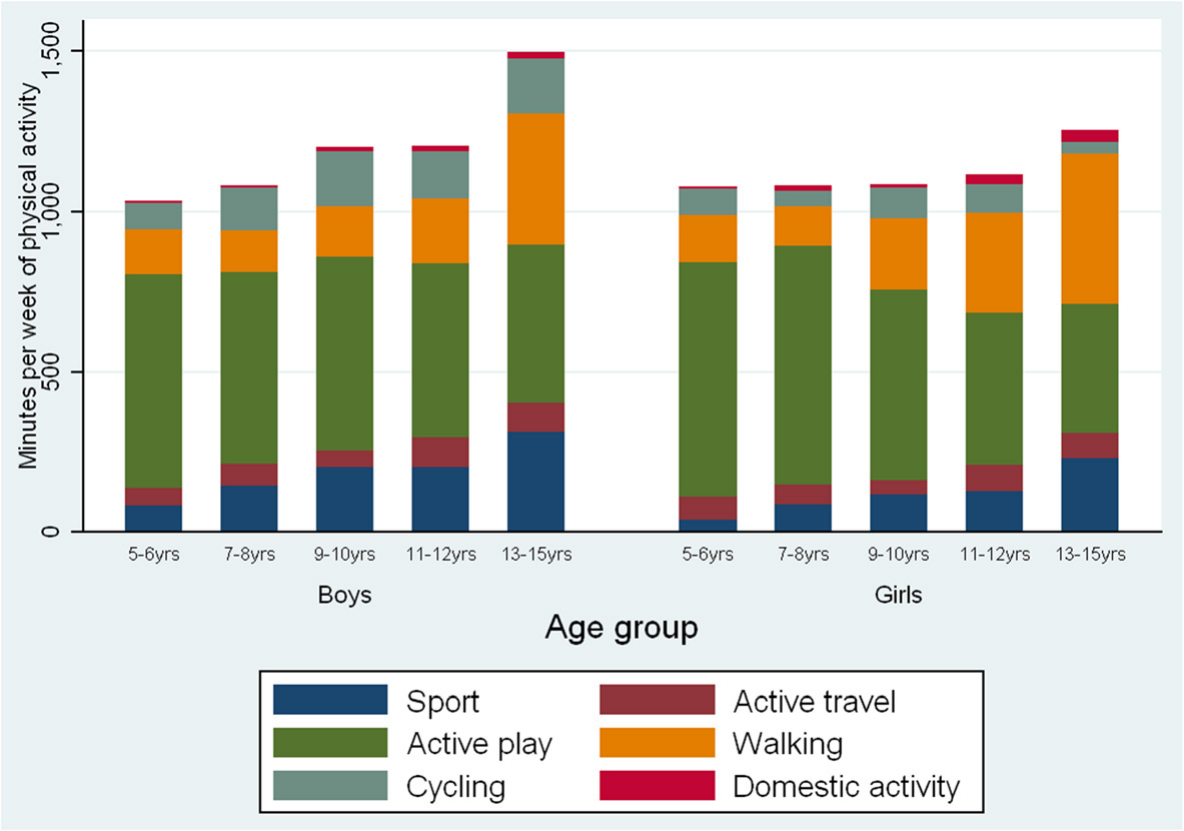
Mean minutes of all physical activity and contributions by each category of activity- by age group and gender in England in 2008.

The main changes in the contributors to overall physical activity with increasing age were a decrease in the proportion contributed from active play (rho = -0.417; p < 0.001) and a corresponding increase in both walking (rho = 0.257; p < 0.001) and sport (rho = 0.219; p < 0.001) (Figure 1; Table 2). Analysis by minutes of activity per week was consistent with these findings (Figure 2). There was a small significant association between age and the proportion of all physical activity achieved through active travel (F = 3.26; p = 0.011) but no significant trend. Though domestic activity contributed a very small proportion to overall physical activity, there was a small but significant increase in this with age (rho = 0.096; p = 0.001).

**Table 2:** Associations of the contributions of activity categories to total activity with age-group, or socioeconomic status

	ANOVA (F)*	**ANOVA (p-value)**	**Spearman’s (rho)**	**Spearman’s (p-value)**
Total minutes of physical activity by age-group (all, n = 1,110)	9.4	<0.001	0.176	<0.001
Total minutes of physical activity by age-group (boys, n = 668)	8.81	<0.001	0.253	<0.001
Total minutes of physical activity by age-group (girls, n = 442)	0.95	0.433	0.035	0.461
Contribution by age-group, % (all)				
Sport†	18.24	<0.001	0.219	<0.001
Active travel	3.26	0.011	0.040	0.180
Active Play†	59.75	<0.001	-0.417	<0.001
Walking†	31.5	<0.001	0.257	<0.001
Cycling	1.83	0.121	0.005	0.871
Domestic activity†	2.45	0.045	0.096	0.001
Contribution by age-group, % (boys)				
Sport†	9.11	<0.001	0.206	<0.001
Active travel	1.36	0.248	0.043	0.271
Active Play†	29.17	<0.001	-0.385	<0.001
Walking†	17.19	<0.001	0.228	<0.001
Cycling	1.78	0.132	0.026	0.508
Domestic activity	0.91	0.456	0.079	0.041
Contribution by age-group, % (girls)				
Sport†	8.33	<0.001	0.195	<0.001
Active travel	2.12	0.077	0.032	0.504
Active Play†	31.41	<0.001	-0.448	<0.001
Walking†	20.61	<0.001	0.348	<0.001
Cycling	2.82	0.025	-0.082	0.084
Domestic activity†	2.67	0.032	0.134	0.005
Contributions in minutes by age-group (all)				
Sport†	19.51	<0.001	0.251	<0.001
Active travel†	6.79	<0.001	0.089	0.003
Active Play†	9.55	<0.001	-0.234	<0.001
Walking†	37.74	<0.001	0.291	<0.001
Cycling	2.5	0.041	0.025	0.416
Domestic activity†	3.46	0.008	0.101	<0.001
Contributions in minutes by age-group (boys)				
Sport†	10.17	<0.001	0.262	<0.001
Active travel†	4.61	0.001	0.117	0.002
Active Play†	2.91	0.021	-0.168	<0.001
Walking†	21.46	<0.001	0.269	<0.001
Cycling	2.84	0.024	0.060	0.121
Domestic activity	1.23	0.295	0.086	0.027
Contributions in minutes by age-group (girls)				
Sport†	8.9	<0.001	0.196	<0.001
Active travel	2.66	0.032	0.039	0.408
Active Play†	8.14	<0.001	-0.329	<0.001
Walking†	22.13	<0.001	0.355	<0.001
Cycling	1.8	0.128	-0.081	0.090
Domestic activity†	3.08	0.016	0.136	0.004
Contribution by IMD, % (all)				
Sport†	10.36	<0.001	-0.191	<0.001
Active travel	0.96	0.427	0.036	0.236
Active Play	2.22	0.650	0.077	0.010
Walking	1.6	0.173	-0.017	0.575
Cycling	0.75	0.560	-0.066	0.029
Domestic activity	0.67	0.611	-0.011	0.708
Contribution by IMD, % (boys)				
Sport†	7.41	<0.001	-0.195	<0.001
Active travel	1.3	0.267	0.011	0.777
Active Play†	2.79	0.026	0.102	0.009
Walking	1.72	0.145	-0.074	0.057
Cycling	1.14	0.334	-0.019	0.632
Domestic activity	0.62	0.652	-0.049	0.205
Contribution by IMD, % (girls)				
Sport†	3.77	0.005	-0.193	<0.001
Active travel	0.57	0.684	0.068	0.155
Active Play	0.43	0.789	0.038	0.426
Walking	1.44	0.219	0.066	0.168
Cycling	1.38	0.241	-0.129	0.007
Domestic activity	0.92	0.455	0.038	0.428
Contributions in minutes by IMD (all)†	5.38	<0.001	0.132	<0.001

†Significant at p < 0.05 for both ANOVA and spearman's.

*Degrees of freedom for all ANOVA tests is 4.

The changes with age were similar in both boys and girls, though the increase in the proportion of all physical activity accounted for by walking was greater among girls. Among boys, the overall increase in total minutes of physical activity with age included an increase in active travel (rho = 0.117; p = 0.002) in addition to walking (rho = 0.269; p < 0.001) and sport (rho = 0.262; p < 0.001). Among girls, though there was no increase in minutes of physical activity with increasing age, mean minutes of walking and sport both increased with age (rho = 0.355 and rho = 0.196; p < 0.001) and mean minutes of active play decreased (rho = -0.329; p < 0.001) (Figure 2; Table 2).

### SES

Sport contributed proportionately less with increasing deprivation (rho = -0.191; p < 0.001) and active play contributed more (rho = 0.077; p = 0.01). Stratified by gender, this trend in contribution of sport remained significant for both boys (rho = -0.195) and girls (rho = -0.193) (p < 0.001 for both) but the increase in active play with increasing deprivation only remained significant for boys (rho = 0.102; p = 0.009).

## Discussion

Overall, this study found significant differences by age, gender and socioeconomic status in the contributors to physical activity among those children who reported doing the recommended amount of physical activity per week. Walking and active play were the biggest contributors to overall physical activity; sport and active school travel contributed a comparatively small proportion each.

Active play was the largest contributor to overall physical activity in younger children but with increasing age walking took over. Such changes with age in the type of activity undertaken by children are not unexpected given their changing maturity across the age range 5–15 years and their increasing independence, particularly given that some of the time spent walking may be for active travel purposes (excluding to/from school). The contribution from sport increased with age for both boys and girls, contributing the most among older boys. A similar pattern of a substantial contribution from walking and a comparatively small contribution to overall physical activity from sport has also been reported in adults in England [21] indicating that this pattern continues into adulthood.

The main differences between the activity profile of girls and boy were the contributions from sport and cycling, which made a smaller contribution among girls than boys (15% combined among girls compared to 28% among boys); and walking and active play which contributed a larger proportion among girls (76% combined compared to 65%). At all ages, sport contributed a larger proportion to physical activity among boys than among girls.

The substantial contributions of walking and active play highlight the important role that informal exercise plays in childrens’ physical activity. This emphasis on informal play activity is also supported by qualitative research which highlights that among young children fun, social interaction and variety, without the element of competition are factors which encourage participation in physical activity [22].

Few other studies have looked at the role of informal play, active travel and domestic activity in addition to structured sport and exercise, in the total physical activity of children and yet this study shows that play in particular is an important contributor. This study is also unique in looking specifically at the contributors to physical activity among *active* children rather than the population as a whole. Limiting the study sample to only active children allows a clearer view of the types of activity that contribute a substantial amount to overall time spent physically active and provides an indication of what types of activity children do when they are achieving the recommended levels of physical activity.

Differences in the activity profile of boys and girls, particularly the role played by sport, may reflect gender differences in motivations for physical activity. Several studies have found that males are more motivated by competition than females [23,24]. Vilhjalmsson and Kristjansdottir [25] concluded, in a study of the gender differences in activity among Icelandic schoolchildren, that the difference in overall physical activity between boys and girls was *entirely* attributable to lower participation in sport among girls. Our study indicates that although the proportion of all activity achieved through sport does differ between boys and girls, other domains of activity such as cycling also contribute to the gender difference in overall physical activity.

Several UK studies have concluded that active school travel does not make a substantial contribution to overall daily physical activity [26-28]; although there is evidence to suggest that active school travel is associated with increased overall physical activity [26,27,29], supporting the ‘activity synergy’ hypothesis. The indication from our analysis, that active school travel contributes only a small amount to total physical activity even among *active* children, further supports these findings. The categorisation of activity data into ‘walking’, ‘cycling’ or ‘active travel to/from school’ does not allow for analysis of the overall contribution of active travel to total physical activity. However, one recent study has shown that non-school active travel is only a small contributor to overall daily moderate to vigorous physical activity in a group of British 8–13 year olds [27].

Our finding that sport contributes less to total physical activity in more deprived children; and that they report less time undertaking sport despite higher overall levels of physical activity, concurs with an Australian study investigating socio-economic position and sports participation which found that adolescents from lower income backgrounds participated in less sport compared with those from wealthier backgrounds [30].

### Study strengths and limitations

Strengths of this study include the use of a large, nationally representative sample. The overall response rate to the HSE (64% of the core and 73% of the boost sample) reduces the likelihood of non-response bias.

Self-reported physical activity can be subject to recall and social desirability bias. However, it remains one of the most practical ways to assess physical activity in a large population survey. The physical activity questionnaire used in the HSE was validated and found to have “moderate validity” for measuring the volume of activity when tested against accelerometry data [18]. Self-reporting is a useful tool for determining the types of activity undertaken therefore this questionnaire is a pragmatic method for measuring the contributors to physical activity in this study. Recall bias, if consistent across respondents and for different categories of activity, is unlikely to affect the comparative analysis by age, gender and socioeconomic status or the relative contributions of categories of physical activity.

A limitation of this study is that curriculum-time school-based physical activity was not captured, based on the assumption that curriculum time physical activity is prescriptive and would vary little across the survey population. This is likely to have led to a small underestimate of total minutes of physical activity. Moreover, intensity of physical activity was not captured on the assumption that all children’s physical activity is at least of moderate intensity. This may have led to an overestimate of total time spent doing ‘at least moderate activity’. Different categories of activity may be more or less at risk of this intensity misclassification than others, introducing bias to our results which cannot be accounted for. However, a recent study of physical activity in British children found that the proportion of physical activity time that was spent at moderate to vigorous intensity when engaged in structured sport, out of home play and non-school active travel (which would include both walking and cycling) was similar [27].

This study adds evidence of the differences in the activities that contribute to total physical activity levels, by age, gender and deprivation. It confirms the importance of informal physical activity for boys and girls of all ages, and highlights the gender differences in adoption of activities such as cycling and sport, even among those children considered active. There is a growing body of qualitative evidence on the barriers and facilitators to physical activity in children that supports the need to target interventions by factors such as age and gender [22,31].

The literature also highlights the important role of environmental facilities for physical activity such as traffic safety; perception of the local environment; and the culture of car use, in influencing physical activity in children [22,31-33]. Knowledge of the types of activities most commonly adopted among children who are active can be used alongside the body of evidence on barriers, motivators and effectiveness of interventions, to help target interventions appropriately and influence the success of strategies to increase physical activity in children at a population level.

## Conclusions

This study provides a greater understanding of the physical activity profile of children who meet the UK physical activity recommendations. This knowledge could help guide future policy decisions to increase physical activity in children and inform effective strategies to increase the proportion of children meeting the physical activity recommendations. In developing interventions, consideration should be given to age, socioeconomic status and gender-related differences in the types of physical activity most likely to be adopted by children. Future research based on objective measures of physical activity behaviours, in combination with qualitative research on the motivations and barriers to specific types of activity will enable a better understanding of what drives physical activity in children.

## Competing interest

The authors have no conflict of interest to declare.

## Authors’ contributions

SP acquired, analysed and interpreted the data, drafted the manuscript and gave final approval of the version to be published. NT designed the study, interpreted the data, critically reviewed the manuscript and gave final approval of the version to be published. CF designed the study, critically reviewed the manuscript and gave final approval of the version to be published.
